# Obstructive Sleep Apnea and the Subsequent Risk of Chronic Rhinosinusitis: A Population-Based Study

**DOI:** 10.1038/srep20786

**Published:** 2016-02-10

**Authors:** Li-Ting Kao, Shih-Han Hung, Herng-Ching Lin, Chih-Kuang Liu, Hung-Meng Huang, Chuan-Song Wu

**Affiliations:** 1 Graduate Institute of Life Science, National Defense Medical Center, Taipei, Taiwan; 2 Sleep Research Center, Taipei Medical University Hospital, Taipei, Taiwan; 3 Department of Otolaryngology, Taipei Medical University Hospital, Taipei, Taiwan; 4 College of Medicine, Fu-Jen University, New Taipei, Taiwan; 5 Department of Otolaryngology, Taipei City Hospital, Taipei, Taiwan

## Abstract

The relationship between obstructive sleep apnea (OSA) and chronic rhinosinusitis (CRS) still remains unclear. This retrospective cohort study aimed to investigate the relationship between OSA and subsequent CRS using a population-based dataset. The study used data from the Taiwan Longitudinal Health Insurance Database 2005. We selected 971 patients with OSA for the study cohort and 4855 patients without OSA for the comparison cohort. Each patient was tracked for 5 years to determine those who were subsequently diagnosed with CRS. Stratified Cox proportional hazard regression analyses were performed to examine the association of OSA with subsequent CRS. The results revealed that 161 (2.76%) of the total sampled patients were subsequently diagnosed with CRS. Subsequent incidences of CRS were found in 64 (6.59%) patients with OSA and 97 (2.00%) patients without OSA. The adjusted hazard ratio (HR) of subsequent CRS for patients with OSA was 3.18 (95% confidence interval: 2.27~4.45) compared to those without OSA. Furthermore, the HR for CRS was similar for subjects with OSA for both genders (with an adjusted HR of 3.44 for males and 2.63 for females). We concluded that patients with OSA had a higher risk of subsequent CRS compared to patients without OSA regardless of sex.

Obstructive sleep apnea (OSA) is a prevalent sleep disorder which is characterized by complete or partial pharyngeal collapse, resulting in snoring, repetitive hypoxia, and brief periods of arousal during sleep^1^,^2^,^3^. Recently, growing biological evidence has supported chronic intermittent hypoxia due to OSA activating inflammatory pathways and increasing oxidative stress, as indicated by high serum levels of C-reactive protein (CRP), interleukin (IL)-1, IL-6, IL-8, and tumor necrosis factor (TNF)-α^4^,^5^,^6^. The systemic inflammatory mechanisms may further develop into endothelial dysfunction, sympathetic excitation, and metabolic dysregulation^1^,^7^,^8^. These pathological processes were found to be etiologically involved in OSA and some chronic diseases^5^,^9^,^10^. Numerous studies have investigated the relationship between OSA and chronic diseases, including cardiovascular diseases, metabolic syndrome, psychosis, etc^11^,^12^,^13^.

CRS is a frequent chronic inflammatory condition involving the nasal mucosa of at least 12 consecutive weeks’ duration^14^. It is a multi-factorial disease, and many extrinsic (environment) and intrinsic (host) factors were indicated to contribute to the incidence and progression of CRS^15^. Extrinsic factors, including microorganism infection, smoking exposure, pollutants, and allergens, may stimulate the adaptive immune system and subsequently lead to CRS^15^,^16^. Abnormal immune responses in CRS may be caused by intrinsic factors or gene-environment interactions^17^. Inflammatory mechanisms which are regulated by the immune system play a key role in the development of CRS.

Nevertheless, although both OSA and CRS may share analogous inflammatory pathophysiological mechanisms, according to our knowledge, no study has attempted to explore the relationship between these two disorders. It still remains unclear whether or not OSA is associated with CRS. Therefore, the aim of this study was to investigate the association between OSA and the subsequent development of CRS using a large population-based dataset in Taiwan.

## Methods

### Database

Administrative claims for this population-based retrospective cohort study were derived from the Taiwan Longitudinal Health Insurance Database 2005 (LHID2005). The Taiwanese National Health Insurance (NHI) program, which was inaugurated in 1995, provides affordable, easily accessible, and comprehensive medical services for all its citizens. The LHID2005 contains longitudinal data on medical claims for 1 million individuals randomly selected from the 2005 Registry of Beneficiaries (*n* = 23.72 million) of the Taiwan NHI program. The high validity of data taken from the Taiwanese NHI program was confirmed by many researchers and by the National Health Research Institutes in Taiwan^18^,^19^.

This study was exempted from full review by the institutional review board (IRB) after consulting with the director of National Defense Medical Center’s IRB, since the LHID2005 consists of de-identified secondary data released to the public for research purposes.

### Study Sample

This study was designed to include a study cohort and a comparison cohort. The study cohort initially included 2955 patients who received a diagnosis of OSA (ICD-9-CM codes 327.23, 780.51, 780.53, or 780.57) during an ambulatory care visit between January 2001 and December 2007. The date of the first diagnosis of OSA was defined as the index date. We then assured that only patients who had been given OSA diagnoses after receiving polysomnography were selected in order to increase the validity of the OSA diagnosis (n = 1636). We further excluded patients under 18 years of age (n = 84) in order to limit the study to the adult population. Furthermore, we also excluded patients who had been diagnosed with a deviated nasal septum (ICD-9-CM 470), allergic rhinitis (ICD-9-CM 477), or CRS (ICD-9-CM 473) (n = 581) prior to the index date. Finally, 971 adult patients with OSA were included in the study cohort.

The matched comparison cohort (*n* = 4855) (five comparison subjects per patient with OSA) was derived from the remaining beneficiaries of the LHID2005. This comparison cohort was selected by matching patients with OSA in terms of sex, age group (18~29, 30~39, 40~49, 50~59, 60~69, 70~79, and ≥80 years), and year of the index date. For the comparison cohort, the year of the index date was simply a matched year in which the comparison subjects had a medical utilization. Additionally, for comparison subjects, the date of their first use of ambulatory care during that matched year was designated the index date. We ensured that none of the selected comparison subjects had received a diagnosis of OSA since the beginning of the NHI program in 1995. We also ensured that none of the comparison subjects had a history of a deviated nasal septum, allergic rhinitis, or CRS prior to their index date.

Each patient was individually tracked for 5 years from their index date to determine all of those who were subsequently diagnosed with CRS.

### Statistical Analysis

All analyses were conducted using the SAS System for Windows (vers. 8.2, SAS Institute, Cary, NC). Chi-squared tests were performed to compare differences between the study and comparison cohorts in terms of monthly insurance salary, geographic location (northern, central, eastern, and southern Taiwan), urbanization level (five levels, with 1 being the most urbanized and 5 being the least), tobacco use disorder (ICD-9-CM 305.1, V1582, 989.84, or 649.0), obesity (ICD-9-CM 278, 278.0, 278.1), hypertension (ICD-9-CM 401~405), hyperlipidemia (ICD-9-CM 272.0~272.4), and diabetes (ICD-9-CM 250). This study used personal monthly health insurance salary as the surrogate variable of monthly income.

A stratified Cox proportional hazard regression analysis was performed to calculate the hazard ratio (HR) for CRS during the 5-year follow-up period between the two cohorts. We present hazard ratios (HRs) along with 95% confidence intervals (CIs). Statistical significance was set at a two-sided *p* value of <0.05.

## Results

The study cohort included 971 patients with OSA and 4855 patients in the matched comparison cohort. The 5826 total patients in the study sample had a mean age of 46.9 ± 14.0 years. The demographic characteristics of patients with and those without OSA are given in Table 1. After matching for gender, age group, and index year, there were significant differences in monthly insured salary (*p* < 0.001), geographic region (*p* < 0.001), urbanization level (*p* = 0.002), tobacco use disorder (*p* < 0.001), obesity (*p* < 0.001), hypertension (*p* < 0.001), hyperlipidemia (*p* < 0.001), and diabetes (*p* < 0.001) between the study and comparison cohorts.

A comparison of the incidences of CRS within 5 years following the index date between the study and comparison cohorts is shown in Table 2. It reveals that 161 (2.76%) of the total sampled patients were subsequently diagnosed with CRS. Subsequent incidences of CRS were found in 64 (6.59%) patients with OSA and in 97 (2.00%) without OSA.

The Cox proportional hazard regression analysis indicated that the crude HR of CRS for patients with OSA was 3.39 (95% CI: 2.47~4.65) compared to those without OSA (Table 2). After adjusting for monthly insured salary, geographical region, and urbanization level, those with OSA were more likely to have been subsequently diagnosed with CRS (HR: 3.18; 95% CI: 2.27~4.45) compared to those without OSA.

The incidence of CRS within 5 years after the index date between patients with OSA and those without OSA stratified by sex is presented in Table 3. It reveals that OSA was significantly related to the subsequent incidence of CRS regardless of sex (with an adjusted HR of 3.44 for males and 2.63 for females).

## Discussion

This population-based retrospective cohort study found that patients with OSA were 3.18-times more likely to be diagnosed with subsequent CRS than were patients without OSA. Both male and female patients with OSA had a higher subsequent risk of CRS compared to those without OSA.

To date, most of the literature has shown that OSA is frequently accompanied with many respiratory symptoms. A previous study explored OSA patients with some accompanying nasopharyngeal conditions, including mucus in the throat, rhinorrhea, a blocked nose, etc^20^. One survey study in Sweden found that respiratory symptoms and diseases, including rhinitis, chronic bronchitis, and asthma, were significantly associated with common symptoms of OSA^21^. Another study in Switzerland reported that perennial allergic rhinitis (having nasal conditions, such as nasal obstruction, runny nose, and sneezing year round and being atopic to at least one perennial allergen) was found in 11% patients with OSA, but only in 2.3% patients with chronic obstructive pulmonary disease^22^. However, no study has attempted to explore the association between OSA and CRS to date.

Although potential mechanisms of the relationship between OSA and CRS remain unclear, it can possibly be explained by the chronic inflammation of OSA. The prior literature documented the association between OSA and inflammation. For example, one study in the US reported that nasal inflammation was frequently present in patients with OSA^23^. They found that some nasal inflammatory indicators including the number of polymorphonuclear leukocytes (PMNs), and concentrations of bradykinin and the vasoactive intestinal peptide (VIP) were significantly higher in patients with OSA than those without OSA. Another study also revealed that chronic intermittent hypoxia due to OSA can trigger inflammatory cytokine release and contribute to mucosal and muscular inflammation of the upper airway^24^. Furthermore, various studies suggested that OSA was highly associated with systemic inflammation^25^,^26^,^27^,^28^. They found that repetitive hypoxia caused by OSA stimulates the production of inflammatory mediators including CRP, IL-1, IL-6, IL-8, TNF-α, etc. These mediators can activate inflammatory pathways and further contribute to endothelial dysfunction, metabolic dysregulation, and sympathetic excitation^7^,^8^. As a result, inflammation may play a major role linking the pathophysiological features of OSA with many chronic diseases including CRS.

Additionally, many studies mentioned that the persistent inflammatory response in CRS might develop from an imbalance of immune interactions between a host and its environment^15^,^16^,^17^. They suggested that elevated expressions of inflammatory mediators might disturb communication and signaling between innate and acquired responses of the immune system^29^,^30^,^31^. Furthermore, abnormal secretion of these mediators might induce an inflammatory response, leading to the migration and activation of eosinophils and neutrophils and a further worsening of the symptoms of CRS^15^,^32^,^33^. Accordingly, it is plausible that abnormal inflammatory responses in patients with OSA might contribute to the development and exacerbation of CRS.

Furthermore, this study showed significant differences in demographic characteristics (including monthly insured salary, geographic region, and urbanization level) prevalence of some co-morbidity between patients with OSA and those without OSA. As for demographic characteristics, the results in our study are consistent with some cross-sectional studies which found that the environment of residence and socioeconomic status would affect the prevalence of OSA^34^,^35^,^36^. As for co-morbidity, many previous studies have demonstrated that OSA is associated with metabolic disorders and cardiovascular diseases. Therefore, it is reasonable that the prevalence of some co-morbidity is higher in patients with OSA than those without OSA in our study^37^,^38^.

The principle strength of this study is the use of a population-based database with universal health benefit coverage in Taiwan. The LHID2005 database provides a sufficient number of subjects to elucidate the relationship between OSA and CRS and increase the statistical power of the results. Moreover, use of this database can also eliminate potential effects of a selection bias.

Nevertheless, there are still several limitations that need to be considered. First, the LHID2005 database used in this study provides no information regarding contact with pollutants or allergens^17^, which are documented as potential risk factors for CRS and might mediate the relationship between OSA and CRS. Therefore, this study used geographic location and urbanization level of the patients’ residence to account for the difference in pollutants or allergens. We have adjusted for geographic location and urbanization level of the patients’ residence in the regression models in order to eliminate the potential impact of the pollutants and allergens. Second, information regarding severity of OSA such as apnea-hypopnea index (AHI) is not available in the LHID2005. Consequently, we could not estimate the potential relationship between OSA severity and the following risk of CRS. Third, the LHID2005 might not contain all patients with OSA or CRS. Some patients with mild symptoms of OSA or CRS might not have sought medical services covered by the NHI program. Finally, most patients selected in our study were of Chinese ethnicity. Therefore, the ability to generalize the results to other racial or ethnic groups is not assured.

## Conclusions

This population-based cohort study found that patients with OSA had a higher risk of subsequent CRS compared to patients without OSA regardless of sex. We recommend that physicians be alert to this relationship and provide routine nasal examinations for patients with OSA. Additionally, physicians can give instructions for patients with OSA to look for appropriate medical services as soon as possible if they have some clinical symptoms about CRS. Nevertheless, further experimental studies are warranted to identify the actual mechanisms for the association between OSA and CRS.

## Additional Information

**How to cite this article**: Kao, L.-T. *et al*. Obstructive Sleep Apnea and the Subsequent Risk of Chronic Rhinosinusitis: A Population-Based Study. *Sci. Rep.* **6**, 20786; doi: 10.1038/srep20786 (2016).

## Figures and Tables

**Table 1:** Demographic characteristics of patients with obstructive sleep apnea (OSA) and comparison group patients (*n* = 5826).

Variable	Patients with OSA *n* = 971		Comparison group*n* = 4855		*p* value
	Total no.	Column %	Total no.	Column %	
Age (years)					1.000
18~29	87	9.0	435	9.0	
30~39	217	22.4	1085	22.4	
40~49	279	28.7	1395	28.7	
50~59	223	23.0	1115	23.0	
60~69	94	9.7	470	9.7	
70~79	57	5.9	285	5.9	
≥80	14	1.4	70	1.4	
Sex					1.000
Male	666	68.6	3330	68.6	
Female	305	31.4	1525	31.4	
Monthly insured salary					<0.001
≤NT$15,840	298	30.7	1549	31.9	
NT$15,841~25,000	302	31.1	1798	37.0	
≥NT$25,001	371	38.2	1508	31.1	
Geographical region					<0.001
Northern	465	47.9	2356	48.5	
Central	264	27.2	1034	21.3	
Southern	230	23.7	1351	27.8	
Eastern	12	1.2	114	2.4	
Urbanization level					0.002
1 (most urbanized)	342	35.2	1557	32.1	
2	288	29.7	1403	28.9	
3	171	17.6	766	15.8	
4	100	10.3	610	12.6	
5 (least urbanized)	70	7.2	86	10.7	
Obesity	77	7.9	49	1.0	<0.001
Tobacco use disorder	43	4.4	119	2.5	<0.001
Hypertension	415	42.8	1224	25.2	<0.001
Hyperlipidemia	393	40.5	1001	20.6	<0.001
Diabetes	188	19.4	614	12.7	<0.001

The average exchange rate in 2011 was US$1.00≈New Taiwan Dollar (NT$)30.

**Table 2:** Prevalences, hazard ratios (HRs), and 95% confidence intervals (CIs) for chronic rhinosinusitis among sampled patients.

Subsequent incidence of chronic sinusitis	Total (*n* = 5826)		Patients with OSA (*n* = 971)		Controls (*n* = 4855)	
	*n*, %		*n*, %		*n*, %	
Yes	161	2.76	64	6.59	97	2.00
No	5665	97.24	907	93.41	4758	98.00
Crude HR (95% CI)	–		3.39^***^ (2.47~4.65)		1.00	
Adjusted HR (95% CI)	–		3.18^***^ (2.27~4.45)		1.00	

Notes: The adjusted HR was calculated by a Cox proportional hazard regression stratified by sex, age group, and index year.

^a^Adjusted for monthly insured salary, geographical region, urbanization level, obesity, tobacco use disorder, hypertension, hyperlipidemia, and diabetes.

^***^p < 0.001.

OSA, obstructive sleep apnea.

**Table 3:** Prevalences, hazard ratios (HRs), and 95% confidence intervals (CIs) for chronic rhinosinusitis among sampled patients according to sex.

Subsequent incidence of chronic sinusitis	Males(*n* = 3996)	Females(*n* = 1830)		Controls(*n* = 1525)
	Patients with OSA(*n* = 666)	Controls(*n* = 3330)	Patients with OSA(*n* = 305)	
	*n*, %	*n*, %	*n*, %	*n*, %
Yes	47 (7.06)	68 (2.04)	17 (5.57)	29 (1.90)
No	619 (92.94)	3262 (97.96)	288 (94.43)	1496 (98.10)
Crude HR (95% CI)	3.56^***^(2.45~5.16)	1.00	2.99^***^(1.64~5.45)	1.00
Adjusted HR (95% CI)	3.44^***^ (2.32~5.10)	1.00	2.63^***^ (1.38~5.01)	1.00

Notes: The adjusted HR was calculated by a Cox proportional hazard regression stratified by age group and the index year.

^a^Adjusted for monthly insured salary, geographical region, urbanization level, obesity, tobacco use disorder, hypertension, hyperlipidemia, and diabetes.

^***^p < 0.001. *OSA, obstructive sleep apnea.*
