# Corrigendum: Obstructive Sleep Apnea and the Subsequent Risk of Chronic Rhinosinusitis: A Population-Based Study

**DOI:** 10.1038/srep46811

**Published:** 2017-09-15

**Authors:** Li-Ting Kao, Shih-Han Hung, Herng-Ching Lin, Chih-Kuang Liu, Hung-Meng Huang, Chuan-Song Wu

Scientific Reports 6: Article number: 20786; 10.1038/srep20786 published online: 02 10 2016; updated: 09 15 2017.

In this Article, Affiliation 3 is incorrectly listed as ‘Department of Otolaryngology, Taipei Medical University Hospital, Taipei, Taiwan’. The correct affiliation is listed below:

Department of Otolaryngology, School of Medicine, College of Medicine, Taipei Medical University, Taipei, Taiwan.

